# Acetamiprid exerts sex-specific effects on adipose tissue of subjects with severe obesity

**DOI:** 10.3389/ftox.2026.1769863

**Published:** 2026-03-10

**Authors:** Giulia Zanchi, Alessia Tammaro, Valentina Monteleone, Rosaria Varì, Carmela Santangelo, Gianfranco Silecchia, Niccolò Petrucciani, Sara Manella, Annalisa Silenzi, Sabrina Tait, Maria Antonietta Ajmone-Cat, Beatrice Scazzocchio, Roberta De Simone, Massimo D’Archivio

**Affiliations:** 1 Centre for Gender-Specific Medicine, Istituto Superiore di Sanità, Rome, Italy; 2 Department of Biomedicine and Prevention, Tor Vergata University of Rome, Rome, Italy; 3 St. Andrea University Hospital, Rome, Italy; 4 National Center for Drug Research and Evaluation, Istituto Superiore di Sanità, Rome, Italy

**Keywords:** inflammation, men, neonicotinoid pesticide, obesity, sex/gender, women

## Abstract

**Background:**

Neonicotinoid pesticides, including acetamiprid (ACE), are widely used in agriculture and pose increasing concerns due to their persistence in the environment and potential human exposure mainly through diet. Available evidence suggests that ACE may disrupt adipocyte function and promote metabolic dysfunctions such as obesity; however, there is limited research on how ACE negatively affects adipose tissue (AT) in men and women. This study utilizes an *ex vivo* translational model to clarify the sex-specific effects of ACE on AT metabolic and inflammatory profile of a vulnerable human substrate, such as the visceral AT of subjects with severe obesity.

**Methods:**

Twenty-four subjects with severe obesity (11 men and 13 women) undergoing bariatric surgery were recruited from St. Andrea University Hospital (Rome, Italy). Visceral adipose tissue biopsies were collected and either treated with ACE or left untreated for further gene and protein expression analysis by RT-qPCR and Western blot, respectively. In addition, adipocytokines secretion, reactive oxygen species production, and free fatty acid release were measured in adipose tissue culture media using commercial or in house assays.

**Results:**

Our findings demonstrate that ACE induces distinct sex-dependent alterations in lipid metabolism, Adipokines regulation, and inflammatory pathways. Specifically, it significantly lowers PPARγ gene expression but raises protein levels, particularly in men. Free fatty acid release increases and Hormone Sensitive Lipase (HSL) drops in both sexes, while Lipoprotein Lipase (LPL) decreases only in women. ACE also promotes inflammation mainly in women, increasing TNF-α, NF-κB, and reactive oxygen species.

**Conclusion:**

These results show that the neonicotinoid ACE worsens AT dysfunction via inflammatory and metabolic pathways in a sex-specific way, likely leading to different risks of obesity-related complications. Overall, these findings provide a mechanistic basis for understanding the toxicological risk of neonicotinoids, highlighting the importance of sex-specific assessment in evaluating metabolic risks of environmental pesticide exposure.

## 1 Introduction

Pesticides are chemical or biological agents primarily used in agriculture to control pests (Sule et al., 2022). Their global use has risen in years due to their high effectiveness in protecting crops. While environmental impacts are a major concern, increasing attention is now focused on human exposure to pesticides. Among pesticides, neonicotinoids like acetamiprid (ACE) are mainly used to control sucking insects such as aphids, whiteflies, and leafhoppers on crops like fruits, vegetables, and ornamentals. Due to its low molecular weight and high-water solubility, ACE can easily penetrate plant tissues and persist for long periods, leading to human exposure mainly through dietary intake (Chen et al., 2014). According to U.S. government analyses, the Food and Drug Administration (FDA) reported, between 2010 and 2015, the presence of neonicotinoids, e.g., imidacloprid and ACE, in various food products, such as fruits, vegetables, tea, water and animal feed (Craddock et al., 2019). Additionally, the European Food Security Agency (EFSA) 2024 statement on the toxicological properties and maximum residue levels of ACE and its metabolites confirms these findings (Hernandez-Jerez et al., 2024). Supporting this, the National Health and Nutrition Examination Survey (NHANES) 2015–2021 study revealed that nearly half of the U.S. population had detectable levels of urinary neonicotinoids, with young children showing even higher exposure levels (Ospina et al., 2019). The main mode of action of neonicotinoids is related to their high affinity for the nicotinic acetylcholine receptors, also in non-target species, including mammals, thus inducing neurotoxicity by alteration of synaptic transmission (Buszewski et al., 2025). However, emerging evidence suggests that neonicotinoids act as metabolism disrupting chemicals (MDCs), therefore exposure to these compounds may be linked to the development of obesity (Wu et al., 2024), since they can disrupt adipocyte function, inducing oxidative stress and contributing to obesity (Sun et al., 2017). Notably, imidacloprid has been shown to stimulate lipid accumulation in 3T3-L1 cells (Mesnage et al., 2018), promoting obesity and insulin resistance in mice (Sun et al., 2017; Sun et al., 2016) and also increasing oxidative stress and lipid accumulation in mice liver (Nimako et al., 2021). Moreover, neonicotinoids can damage DNA, lipids, and proteins, likely through the increase of reactive oxygen species (ROS) (Wang et al., 2018). Recent evidence suggests pesticides are implicated in disrupting key molecular pathways that govern adipose tissue (AT) differentiation, appetite regulation, and metabolic homeostasis (Martyniuk et al., 2022). Overall, this disruption may facilitate the onset of obesity and related metabolic and endocrine diseases (D’Archivio et al., 2024). Obesity is a growing global public health challenge and one of the leading causes of death worldwide (WHO, 2025). The multifactorial nature of obesity makes prevention and treatment particularly challenging, requiring comprehensive strategies that address these interconnected biological, social, and policy factors. Obesity is characterized by dysfunctional visceral AT that secretes pro-inflammatory adipokines, impairing glucose and lipid metabolism as well as adipogenesis and lipolysis (Romacho et al., 2014; Močnik and Varda, 2021). This dysfunction contributes to the establishment of chronic low-grade inflammation (Russo et al., 2021), which increases the risk of obesity-related disorders, such as type 2 diabetes mellitus (T2DM), cardiovascular diseases, and certain cancers (Nadal et al., 2017). There is growing evidence highlighting significant sex and gender differences in obesity development, clinical manifestations, and associated risk factors. Indeed, the physiological and hormonal differences between men and women lead to variations in fat distribution, metabolic responses, and susceptibility to obesity-related complications (Koceva et al., 2024; Muscogiuri et al., 2024). However, few studies have examined how exposure to MDCs interacts with sex and gender in the development of obesity. Some findings suggest that MDC exposure increases body mass index (BMI) in men but decreases it in women (Elobeid et al., 2010), while other studies report decreased waist circumference in males but increased waist circumference in females (Kershaw and Flier, 2004). These differences might be due to hormonally driven fat distribution patterns, with women more prone to hip fat accumulation and men to abdominal fat (Buszewski et al., 2025). Despite these findings, to the best of our knowledge, limited research has investigated how neonicotinoids, particularly ACE, can impact AT. This study is important considering the central role of AT dysfunctions in contributing to the risk of obesity-related disorders. In this context, it is also crucial to consider sexual dimorphism characterizing AT physiology. Since neonicotinoids like ACE can act as MDCs, they may interact differently with these sex-specific biological backgrounds. Understanding these distinct mechanisms is fundamental for a precise risk assessment, as the same environmental exposure might lead to divergent metabolic outcomes depending on biological sex. This study, conducted within the framework of the Health Extended Alliance for Innovative Therapies, Advanced Lab-research, and Integrated Approaches of Precision Medicine (HEAL ITALIA) partnership, aims to elucidate the mechanisms of action of ACE on AT in severely obese patients undergoing bariatric surgery, with a particular focus on sex-specific responses.

## 2 Materials and methods

### 2.1 Subjects' recruitment and sample collection

The study has been approved by the National Ethics Committee (Istituto Superiore di Sanità AOO-ISS-03/05/2023-0020590 Class: PRE BIO CE 01.00) and conducted in accordance with the “Declaration of Helsinki”. Subjects participated in the study on a voluntary basis. Interested subjects were provided with information on the study description and requested consent to participate and take a sample of adipose tissue. ‘Clinical trial number: not applicable.’

Twenty-four subjects with severe obesity (men/women ratio 1:1) were recruited from the complex Operational Unit of General and Hepatobiliary Surgery of St. Andrea University Hospital (Rome, Italy) (Table 1). Subjects were recruited consecutively over a 10-month period (from September 2024 to June 2025). The sample size was determined by the availability of eligible patients meeting the inclusion criteria during this timeframe, which represents a common approach for *ex vivo* studies involving human surgical biopsies. The two study populations (male and female) are homogeneous in terms of environmental factors exposure: 70% of individuals live in the same geographical area, and they exhibit similar distributions for employment status and smoking status history.

**TABLE 1:** General parameters of twenty-four subjects with severe obesity. Comparison between men (11) and women (13). Age, BMI, Waist Circumference, Hip Circumference, insulinemia, Glycemia, HOMA-IR, HB Glycate, PCR, Total cholesterol, HDL, LDL and triglycerides are presented as mean ± SD. Significance was assessed using an independent samples t-test. Menopause, hypertension, dyslipidemia, hepatic steatosis and smoking state are presented as subjects affected/subject total (%). Significance was assessed using a Fisher test. BMI = Body Mass Index.

General parameters	Men (11) Mean ± SD	Women (13) Mean ± SD	p-value (* = 0.05)
Age	49.6 ± 15.4	51.6 ± 11.6	0.74
BMI	45.6 ± 3.1	39.9 ± 5.8	0.23
Waist Circumference	133 ± 22.2	113 ± 14.5	0.65
Hip circumference	130 ± 17	131 ± 18.4	0.10
Insulinemia uU/mL	12.8 ± 5.00	13 ± 6.4	0.93
Glycemia mg/dL	100.6 ± 27.8	98 ± 13.8	0.78
HOMA-IR	2.8 ± 1.7	2.7 ± 2	0.95
HB Glycate %	8 ± 7.6	5.6 ± 0.5	0.32
PCR mg/dL	8.2 ± 11	5.2 ± 4.8	0.44
Total cholesterol mg/dL	168.5 ± 36	170.3 ± 34	0.9
HDL mg/dL	40.3 ± 9.6	46.7 ± 12.7	0.17
LDL mg/dL	115.4 ± 31.4	110.9 ± 30.9	0.72
Triglycerides mg/dL	103 ± 42.7	92.8 ± 47.3	0.58
Menopause % subjects affected/total subjects	—	54%	—
Hypertension % subjects affected/total subjects	36.36%	46.15%	0.69
Dyslipidemia % subjects affected/total subjects	27.27%	7.69%	0.3
Hepatic steatosis % subjects affected/total subjects	36.36%	63.64%	1
Smoking state % subjects affected/total subjects	63.64%	46.15%	0.23

Subjects were selected based on the following inclusion and exclusion criteria:

Inclusion criteria:

- Age range 21-75;
- Severe obesity (BMI >30);
- Bariatric surgery.

Exclusion criteria:

- COVID positivity in the last 6 weeks;
- Clinical evidence of active infection;
- Chronic diabetics;
- Recent use (within 14 days) of antibiotics or anti-inflammatory drugs; radiotherapy; chemotherapy; steroid or cortisone anti-inflammatory therapies;
- Drug or alcohol abuse;
- Chronic kidney disease;
- Neoplastic diseases;
- Pregnancy;
- Mental disability.

During the first clinical visit, approximately 2 weeks prior to surgery, the subjects' height, weight, and waist circumference were measured to calculate their body mass index (BMI). Visceral AT biopsies were collected during bariatric surgery. Immediately following excision, tissue samples were placed in a sterile physiological saline solution (0.9% NaCl) to ensure tissue viability and integrity during transport to the laboratory. The biological samples were immediately shipped to Istituto Superiore di Sanità via a specialized courier under conditions that guaranteed the correct preservation and integrity of the samples.

### 2.2 Acetamiprid working solution preparation

ACE was purchased as a powder (CAS 135410-20-7, Supelco, Germany, 100 mg, purity ≥98%) and dissolved in DMSO (Dimethyl sulfoxide) (Sigma-Aldrich, Milan, Italy) in a glass amber vial to obtain a 10 mM stock solution stored at −20 °C, ensuring more stability and repeatability. The desired final concentration of 150 nM is obtained on the day of treatment by diluting the stock concentration in culture medium.

### 2.3 Experimental conditions

To define the experimental conditions, we carried out preliminary trials incubating AT with different concentrations of ACE (from 1 to 200 nM) for different times (6, 12, 24 and 48 h). Given the critical role of PPARγ in adipogenesis and in maintaining the phenotype of mature adipocytes (Sun et al., 2021), we analysed the dose–response curve of ACE on PPARγ gene expression. The threshold for selecting the optimal concentration was the lowest dose of ACE producing a statistically significant reduction in PPARγ expression compared to untreated tissues (data not shown). This analysis led us to the selection of 150 nM as the minimum effective concentration of ACE required after 24 h of incubation. This concentration falls in the EFSA acceptable daily intake (ADI) and acute reference dose (ARfD) of 0.005 mg/kg body weight (per day) (Hernandez-Jerez et al., 2024).

### 2.4 Sample preparation

Immediately after the arrive at the laboratory, AT biopsies were placed in a sterile Petri dish containing physiological saline solution (0,9% NaCl) and sectioned into homogenous fragments of approximately 200 mg and transferred into a Corning Falcon 6-well Cell Culture Plate. Each well contained 2 mL of DMEM supplemented with antibiotics (100 U/mL penicillin and 100 μg/mL streptomycin) and 1% stable glutamine. The multi-well plates were incubated for 24 h in a 5% CO_2_ humidified atmosphere, with ACE 150 nM or untreated (CTR) at 37 °C.

### 2.5 Preparation of adipose tissue homogenates and Western blot

Tissue lysates for Western blotting were prepared as described by Ormazabal et al. (2018). Tissue homogenization was performed using a specific lysis buffer containing protease and phosphatase inhibitors. Protein quantitation was performed by Lowry method (Bio-Rad, Hercules, CA, United States) (Lowry et al., 1951).

12 µg of lysates from AT were then boiled with Laemmli sample buffer for 5 min, resolved by 4%- 20% SDS-PAGE, and transferred onto nitrocellulose membranes (Invitrogen, United States). Immunoblotting analyses were carried on the whole extracts using specific polyclonal antibodies for PPARγ, phospho-p65 NF-κB, NF-κB (ELK Biotechnology Co., Ltd., United States). Blots were treated with appropriate anti-rabbit and anti-mouse secondary antibodies (BIOSS, United States) conjugated with horseradish peroxidase, followed by ECL detection (Amersham Bio-Sciences, Buckinghamshire, United Kingdom). Equal loading of proteins measured by Lowry method was verified by immunoblotting with a mouse anti-Cyclophilin antibody (Sigma-Aldrich, United States). Densitometric analysis was performed using a molecular imager FX (Bio-Rad, Hercules, CA, United States).

### 2.6 RNA extraction and cDNA synthesis from adipose tissue

Total RNA extraction was performed with TRIzol reagent (Invitrogen, United States) following the manufacturer’s instructions, with minor modifications to account for tissue density and lipid content. RNA concentration and purity were determined spectrophotometrically (NanoDrop ND-1000, Thermo Scientific), and only samples with A260/A280 ≥ 2 were used for downstream analyses. cDNA was synthesized from 1 µg of total RNA using the OptiFast cDNA Synthesis Kit (Meridian Bioscence) following the manufacturer’s instructions.

### 2.7 Real-Time PCR

Gene expression in adipose tissue cDNA was analysed by qPCR. Specific primers for Leptin, Adiponectin, PPARγ, TNF-α, HSL (Hormone-Sensitive Lipase) and LPL (Lipoprotein Lipase) were used and synthesized by Life Technologies (Table 1, supplementary data). Glyceraldehyde-3-phosphate dehydrogenase (GAPDH) was used as housekeeping gene to normalize expression levels. qPCR reactions were performed in duplicate using the SensiFAST SYBR No-ROX Kit (Meridian Bioscence, United States) on a Bioer 9600 thermal cycler (Bioer Technology Co. Ltd., Hangzhou, CN), following the manufacturer’s instructions. Relative expression of target genes was determined using the 2^−ΔΔCT^ method, considering the threshold cycle (Ct) of the sample relative to the internal reference GAPDH (ΔCt) and to the untreated sample (ΔΔCt).

### 2.8 Adipocytokine measurements

Adiponectin and leptin secretion was assessed in AT culture media using commercial ELISA kits: Human ADP Adiponectin (ELK Biotechnology Co., Ltd., United States); Human LEP Leptin (ELK Biotechnology Co., Ltd., United States) according to the manufacturer’s instructions. Samples and standards were loaded in duplicate into 96-well plates, and the absorbance was measured by the VICTOR Nivo Multimode Microplate Reader at 450 nm (Perkin Elmer). The unknown concentrations in samples were derived using the standard curve of each adipocytokine and the software GraphPad Prism 8.0 (GraphPad Software Inc.).

### 2.9 Quantification of reactive oxygen species

ROS levels in AT culture media were determined by a spectrofluorometric method using the 2′-7′-dichlorofluorescein-diacetate (H2DCFDA) dye (Invitrogen, United States), following the protocol previously described by Sengupta et al. (Sengupta et al., 2019). Fluorescence was measured using the VICTOR Nivo Multimode Microplate Reader with excitation wavelength of 485 nm and emission wavelength of 530 nm. ROS levels were expressed as relative fluorescence units.

### 2.10 Free fatty acid release

Free fatty acid (FFA) release was measured in AT culture media using a colorimetric assay kit (Sigma-Aldrich, United States) according to the manufacturer’s instructions. Samples and standards were loaded in duplicate, and the absorbance was measured by VICTOR Nivo Multimode Microplate Reader at 450 nm. FFA concentrations were calculated from standard curves of palmitic acid and results were expressed in µM.

### 2.11 Statistical analyses

Statistical analyses were conducted using GraphPad Prism 8 (GraphPad Software Inc., San Diego, CA, United States). GraphPad Prism 8 was also utilized for all graphical design. Adiponectin, leptin, ROS and FFA supernatant levels results were presented as mean ± standard error (SEM). Gene expression data were presented as 2^−ΔΔCT^ mean ± SEM. Western blot analysis results were presented as fold increase relative to the control group, which is arbitrarily set to 1. Data were analysed separately for men and women, comparing treated samples with corresponding control samples. A t-test analysis was performed. Differences among groups were considered statistically significant if the p-value was ≤0.05.

## 3 Results

### 3.1 ACE induces a proinflammatory environment in adipose tissue in a sex-specific manner

**FIGURE 1:**
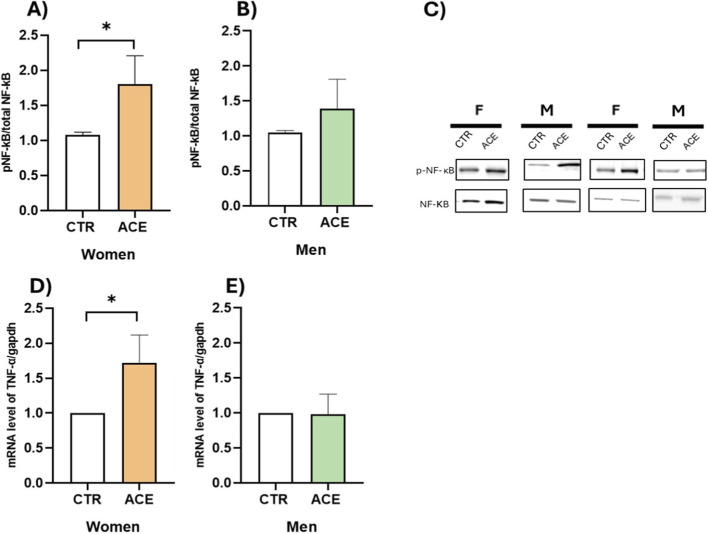
ACE effects on inflammatory pathways. Phospho (p)-NF-κB protein expression in visceral adipose tissue (AT) of women **(A)** and men **(B)** subjects, treated or not with ACE 150 nM, by Western blot. Protein expression is presented as a fold increase relative to the control group, which is arbitrarily set to 1. Representative images of Western blot analysis of total NF-kB and p-NF-kB are shown **(C)**. TNF-α gene expression in visceral AT of women **(D)** and men **(E)** subjects, treated or not with ACE 150 nM, by Real-Time PCR. Gene expression is presented as 2^−ΔΔCT^ mean ± SEM. Statistical significance was calculated by t-test. Differences among groups were considered statistically significant if the p-value was ≤0.05.*p ≤ 0.05. CTR, untreated; ACE, acetamiprid 150 nM.

**FIGURE 2:**
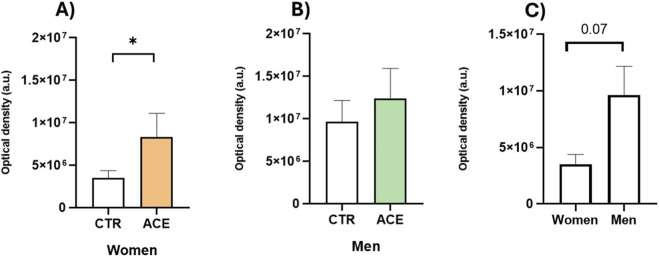
*ACE effects on ROS production.* Reactive oxygen species (ROS) release measured in visceral adipose tissue (AT) culture media by spectrofluorometric method using the 2′-7′-dichlorofluorescein-diacetate (H2DCFDA) dye. ROS contents in AT culture media of women **(A)** and men **(B)** subjects treated or not with ACE. Basal ROS content in AT culture media of women and men **(C)**. Results are expressed as mean ± SEM of arbitrary unit (a.u.). Statistical significance was calculated by t-test. Differences among groups were considered statistically significant if the p-value was ≤0.05. *p ≤ 0.05. CTR, untreated; ACE, acetamiprid 150 nM.

To investigate inflammatory mechanisms in AT of obese subjects, we first examined the role of ACE in NF-κB protein activation, a key regulator of inflammation. The results demonstrated an enhanced proinflammatory response in AT following ACE treatment, with a statistically significant effect observed in women (Figures 1A–C; p = 0.05). The NF-κB signalling pathway is the principal mediator through which members of TNF cytokine family, such as TNF-α, rapidly induce the transcription of genes essential for the inflammatory response (Hayden and Ghosh, 2014). To further explore this, we examined TNF-α gene expression, finding that ACE treatment significantly increases TNF-α expression only in women (Figures 1D,E; p = 0.05). Regarding ROS production in the culture media of ACE-stimulated AT, we observed an increase in both sexes, although only in women the increase was statistically significant (Figures 2A,B; p < 0.05). It is worth noting that the basal release of ROS from AT differed between sexes, with men exhibiting higher production than women (Figure 2C; p = 0.07). These findings collectively suggest a sex-specific modulation of oxidative stress and inflammatory pathways by ACE in AT.

### 3.2 ACE differentially modulates Adipokines in adipose tissue from women and men subjects

**FIGURE 3:**
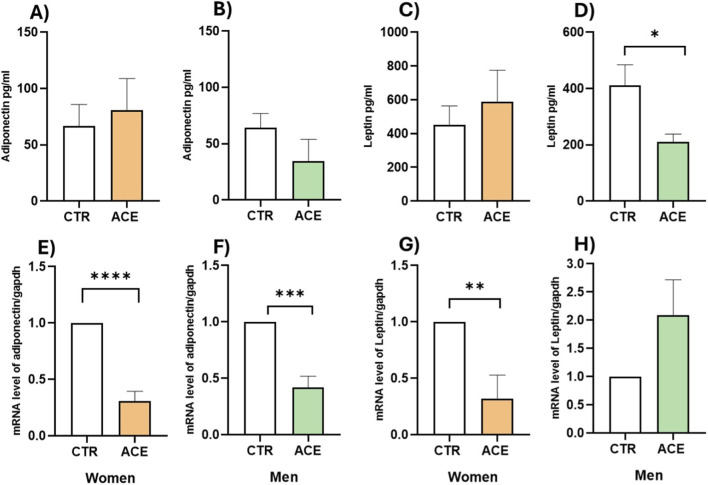
*ACE effects on adipocytokines.* Adiponectin release in AT culture media of women **(A)** and men **(B)** subjects, treated or not with ACE 150 nM, by Enzyme-Linked Immunosorbent Assay (ELISA). Leptin release in AT culture media of female **(C)** and male **(D)** patients, treated or not with ACE 150 nM, by Enzyme-Linked Immunosorbent Assay (ELISA). Results are expressed in pg/mL of mean ± SEM. Adiponectin gene expression in visceral adipose tissue (AT) of women **(E)** and men **(F)** subjects, treated or not with ACE 150 nM, by Real-Time PCR. Leptin gene expression in visceral AT of female **(G)** and male **(H)** patients, treated or not with ACE 150 nM, by Real-Time PCR. Gene expression is presented as 2^−ΔΔCT^ mean ± SEM. Statistical significance was calculated by t-test. Differences among groups were considered statistically significant if the p-value was ≤0.05. ****p ≤ 0.0001; ***p ≤ 0.001; **p ≤ 0.01. CTR, untreated; ACE, acetamiprid 150 nM.

Consequently, we proceeded to evaluate the effect of ACE on Adipokines. In women, ACE treatment induced a non-significant increase in the release of both adiponectin (Figure 3A) and leptin (Figure 3C) in the AT culture media. Conversely, in men an opposite effect was noted, with a reduction in the release of both Adipokines, significant only for leptin levels (Figures 3B–D; p < 0.05). Gene expression analysis further revealed that ACE significantly decreased the expression of both adiponectin (Figure 3E; p < 0.0001) and leptin (Figure 3G; p < 0.01) in the AT of women. In men, ACE treatment differently affected adipocytokines mRNA expression, resulting in not significant increase in leptin (Figure 3H) and a statistically significant decrease in adiponectin (Figure 3F; p < 0.001). These findings provide novel insights, demonstrating a significant sex-specific effect of ACE exposure on Adipokines levels, which may contribute to differential metabolic outcomes between men and women.

### 3.3 Sex-specific effects of ACE on lipid metabolism in adipose tissue

**FIGURE 4:**
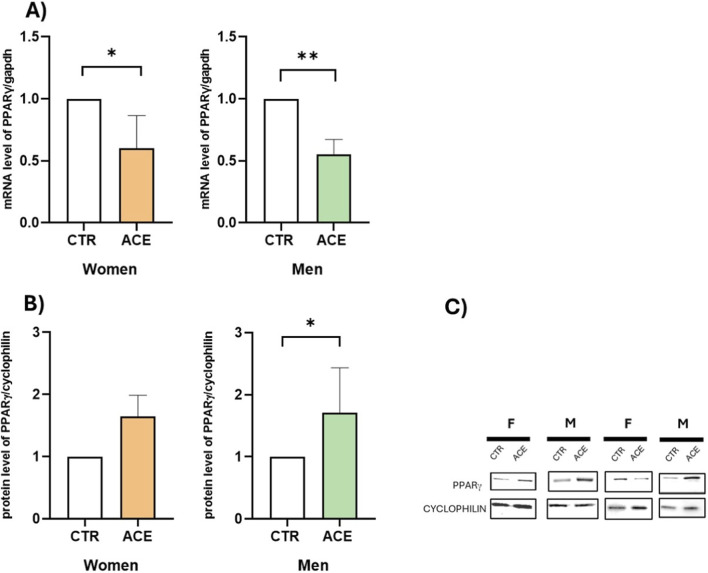
*ACE effects on PPARγ*. PPARγ gene expression in visceral adipose tissue (AT) of women and men subjects, treated or not with ACE 150 nM, by Real-Time PCR **(A)**. Gene expression is presented as 2^−ΔΔCT^ mean ± SEM. PPARγ protein expression in visceral AT of women and men subjects, treated or not with ACE 150 nM, by Western blot **(B)**. Protein expression is presented as fold increase relative to the control group, which is arbitrarily set to 1. Representative image of Western blot analysis of PPARγ and Cyclophilin is shown **(C)**. Statistical significance was calculated by t-test. Differences among groups were considered statistically significant if the p-value was ≤0.05. *p ≤ 0.05; **p ≤ 0.01. CTR, untreated; ACE, acetamiprid 150 nM.

**FIGURE 5:**
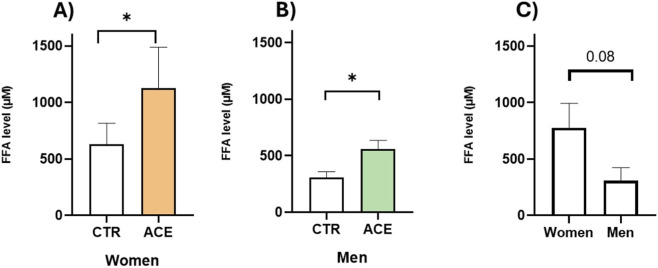
*ACE effects on FFAs release*. FFAs release in AT culture media from women **(A)** and men **(B)** subjects treated or not with ACE 150 nM. Basal FFAs release in women and men **(C)**. Results are expressed as mean ± SEM. Statistical significance was calculated by t-test. Differences among groups were considered statistically significant if the p-value was ≤0.05. *p ≤ 0.05. CTR, untreated; ACE, acetamiprid 150 nM.

**FIGURE 6:**
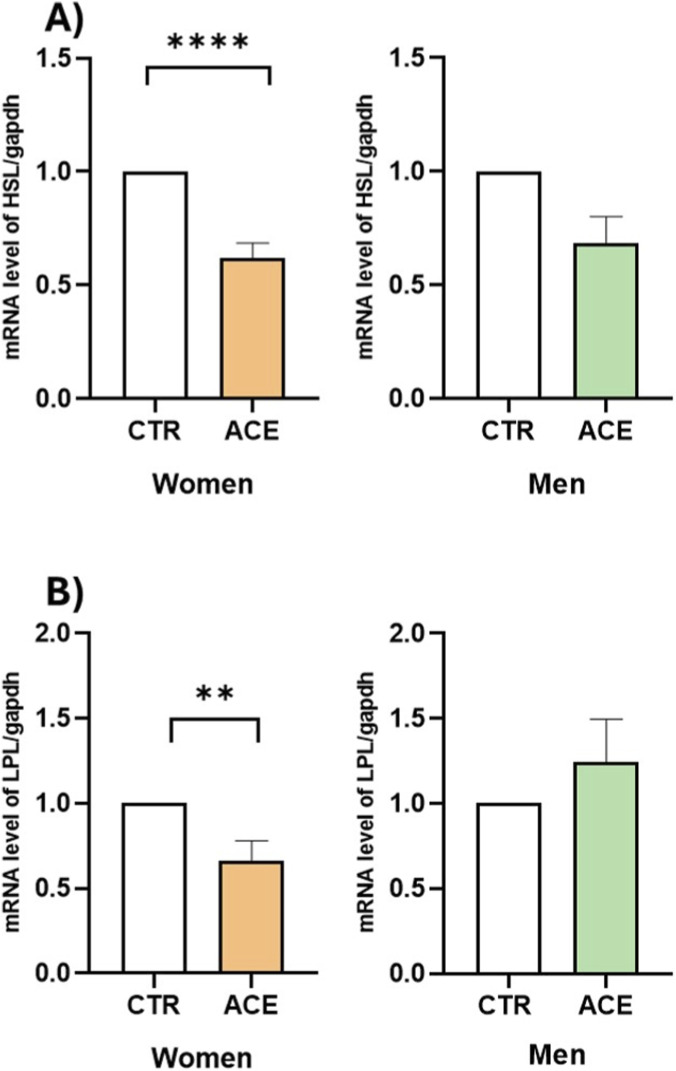
*ACE effects on HSL and LPL gene expression.* HSL **(A)** and LPL **(B)** gene expression in visceral adipose tissue (AT) of women and men subjects, treated or not with ACE 150 nM, by Real-Time PCR. Gene expression is presented as 2^−ΔΔCT^ mean ± SEM. Statistical significance was calculated by t-test. Differences among groups were considered statistically significant if the p-value was ≤0.05. ****p ≤ 0.0001; **p ≤ 0.01. CTR, untreated, ACE acetamiprid 150 nM.

To investigate this effect, we examined mRNA and protein expressions of PPARγ, the master regulator of adipocyte differentiation and fat accumulation, in AT from male and female obese subjects. The results demonstrated that ACE significantly downregulated PPARγ gene expression in both sexes (Figure 4A, p < 0.05; p < 0.01). Interestingly, ACE significantly upregulated PPARγ protein expression (Figures 4B,C), with a more pronounced and statistically significant effect observed in men (p < 0.05). To further explore the mechanism by which ACE influences lipid metabolism, FFA production was measured in AT culture media. The results revealed that ACE stimulation significantly increased FFA secretion in both sexes (Figures 5A,B; p < 0.05), despite the lower basal FFA release observed in males (Figure 5C; p = 0.08). The expression of HSL and LPL, two key genes involved in lipid metabolism, was then examined. Notably, ACE treatment significantly downregulated HSL (Figure 6A; p < 0.0001) and LPL (Figure 6B; p < 0.01) gene expression, only in women, suggesting a sex-dependent effect of this compound on lipid metabolism impairment.

## 4 Discussion

In this study, we investigated whether the effects of the neonicotinoid ACE on AT differ between obese men and women. We also explored the underlying mechanisms by focusing on inflammatory and metabolic alterations. The widespread use of pesticides has led to their pervasive presence in the environment, posing risks to non-target organisms, including humans (Costas-Ferreira and Faro, 2021). Due to their high toxicity and potential for bioaccumulation, organophosphate and organochlorine pesticides have been gradually phased out over recent decades (Mostafalou and Abdollahi, 2017; Ataei and Abdollahi, 2022). Neonicotinoids are mainly used on vegetables and fruits, and because of their low molecular weight and high-water solubility, they can easily penetrate plant tissues and persist for a long time (Chen et al., 2014). The main route of human exposure to these pesticides is therefore through diet and they may disrupt the normal function of white, brown, and beige AT, thereby promoting obesity and related metabolic diseases such as T2DM (Gutgesell et al., 2020).

Taken together, these factors make the effects of neonicotinoids on human health a significant concern, especially considering that chronic or early-life exposure to endocrine disruptors has been linked to numerous adverse health outcomes, including obesity (Asteria et al., 2024). Our study offers new insights into the effects of neonicotinoid ACE on AT from male and female patients with severe obesity. Sex-related differences in body composition are well established. Women generally have a higher percentage of body fat, whereas men tend to accumulate fat centrally in visceral (android) depots and women predominantly in peripheral (gynoid) depots. The greater visceral adiposity observed in men is associated with worse metabolic outcomes compared to the female pattern, which is less strongly linked to metabolic risk (Muscogiuri et al., 2024). However, women seem particularly vulnerable to pesticides exposure due to biological factors such as higher AT mass and hormonal fluctuations during pregnancy, lactation, and menopause, as well as gender-specific roles and exposure patterns (Atinkut et al., 2022). To date, only a few human studies have investigated these differences, and none has focused specifically on sex-specific responses. Our findings clearly demonstrate that ACE induces a proinflammatory environment in AT of obese subjects, with sex-specific effects mediated by upregulation of TNF/NFκB pathways. This aligns with existing evidence from the literature on the key role of TNF-α and its signaling pathways in AT inflammation and metabolism (Varra et al., 2024). TNF-α is a key cytokine in systemic inflammation, playing a central role in chronic inflammation associated with obesity. In this condition, it is overproduced by hypertrophic adipocytes and infiltrating macrophages, thereby contributing to insulin resistance and metabolic dysregulation (Cawthorn and Sethi, 2008). TNF-α regulates the transcription of inflammatory genes, including those in the NF-κB pathway, and promotes the production of ROS, consistent with our findings that ACE stimulates NF-κB activation and ROS production, particularly in women.

Men and women differ in the oxidative stress and inflammation mechanisms. Our data are consistent with the findings of Martínez de Toda et al. (2023), indicating that men exhibit greater ROS production, higher basal inflammation, but a weaker response to stimuli than women do. In contrast, women show more robust immune and inflammatory responses upon stimulation (Martínez de Toda et al., 2023). Another study also indicates that TNF-α inhibits fatty acid uptake and lipogenesis by repressing PPARγ (Cawthorn and Sethi, 2008), the master regulator of adipocyte differentiation and lipid storage, findings that are consistent with the observed effects in our study. Indeed, ACE reduced PPARγ mRNA levels in both sexes, while the concomitant increase in PPARγ protein levels through post-transcriptional dysregulation, may reflect a compensatory mechanism to limit excessive FFA release, given the well-established role of PPARγ in promoting adipogenesis.

The increased secretion of FFAs observed in both sexes after ACE treatment confirms the capacity of ACE to disrupt metabolism and implies that elevated circulating FFAs may contribute to insulin resistance and obesity (Pérez-Bermejo et al., 2024). The uncontrolled FFA release was associated with altered expression of HSL and LPL, key enzymes regulating FA mobilization and deposition in AT (Althaher, 2022). Specifically, the marked downregulation of HSL expression in both sexes, combined with a selective reduction in LPL expression in females, indicates disrupted lipid metabolism mediated by sex-specific regulatory mechanisms (Pujol et al., 2003). The mechanistic basis for the sex-specific response to ACE likely resides in the complex interplay between environmental triggers and the distinct physiological landscapes of men and women. Sexual dimorphism in visceral AT is largely driven by sex steroids (Steiner and Berry, 2022; Lee et al., 2013); ACE, acting as an MDC, may interfere with estrogen receptor signaling, leading to the observed downregulation of LPL compared to men. Furthermore, the exacerbation of oxidative stress observed in our female cohort suggests that ACE might differentially impair sex-linked antioxidant defense systems or mitochondrial function. These biological differences may render the visceral AT of one sex potentially more vulnerable to the toxicological insult of neonicotinoids, shifting the metabolic balance toward a more severe dysfunctional state.

In morbidly obese subjects, AT function is severely compromised, and insulin resistance impairs adipocyte responsiveness to insulin, reducing suppression of lipolysis (Gastaldelli et al., 2017). Interestingly, our data demonstrate that AT from subjects with severe obesity represents a vulnerable substrate where ACE exacerbates pre-existing lipid metabolism dysfunctions. Indeed, our results suggest that ACE significantly aggravates this metabolic impairment by disrupting the already compromised lipolytic pathway (Althaher, 2022). Adipocytokines such as adiponectin and leptin also play critical roles in AT dysfunction associated with obesity (Frühbeck et al., 2018). Under physiological conditions, their production is tightly regulated, but obesity disrupts these mechanisms (Frühbeck et al., 2018), and neonicotinoid exposure can further exacerbate this impairment (Pérez-Bermejo et al., 2024). Our findings reveal that ACE affects adiponectin and leptin secretion and gene expression in a sex-dependent manner. Leptin promotes satiety and increases energy expenditure, while adiponectin enhances insulin sensitivity and reduces inflammation. Obesity typically features elevated leptin and reduced adiponectin levels (Frühbeck et al., 2018). Based on our findings, it can be hypothesized that women may initially mount a compensatory response to ACE-induced disruptions by adjusting adipokines secretion, an adaptive response not observed in men. However, the reduced gene expression of both leptin and adiponectin in women suggests that this compensatory mechanism may be insufficient. In men, although adiponectin gene is similarly reduced, ACE exposure boosted leptin gene expression in AT, possibly reflecting a compensatory response alongside a potential increase in leptin resistance.

The present study has several limitations that must be acknowledged. First, the sample size was limited, and a larger cohort is necessary to draw more robust conclusions. Furthermore, we did not assess if the observed effects were due to the parent compound and/or also to metabolite(s). Finally, the lack of baseline measurement of ACE levels already present in the AT biopsies. Environmental background levels could influence the tissue’s initial metabolic state. Nevertheless, we addressed this by employing an *ex vivo* model where each sample served as its own internal control, ensuring that the reported effects are specifically induced by the experimental ACE treatment. Future studies incorporating biomonitoring through LC-MS/MS could further refine our understanding of how pre-existing body burdens interact with acute pesticide exposure.

## 5 Conclusion

In summary, our study demonstrates that ACE affects AT functions in obese subjects by exacerbating a pro-inflammatory and metabolically dysregulated state, with clear sex-specific effects. These alterations likely involve TNFα-mediated modulation of adipocytokines biology within the obese milieu. Given the significant disruption of lipid metabolism and inflammation pathways, particularly in individuals with severe obesity, our findings underscore the importance of considering sex differences when evaluating the metabolic risks associated with pesticide exposure. Further research is warranted to confirm our findings with larger studies, deepen our understanding of the long-term health implications and to develop sex-specific strategies for mitigating the adverse effects of ACE on AT function and metabolic health.

## Data Availability

The datasets presented in this study can be found in online repositories. The names of the repository/repositories and accession number(s) can be found in the article/Supplementary Material.
