# Supplementary material for: Acetamiprid exerts sex-specific effects on adipose tissue of subjects with severe obesity
Source: Front Toxicol. 2026 Mar 10;8:1769863. doi: 10.3389/ftox.2026.1769863 (PMC13008313; doi:10.3389/ftox.2026.1769863)
Supplement: Supplementary file 2 [file Table1.docx]

| **General Parameters** | **Men (11)**  **Mean ± SD** | **Women (13)**  **Mean ± SD** | **p-value**  **(*=0.05)** |
| --- | --- | --- | --- |
| Age | 49.6±15.4 | 51.6±11.6 | 0.74 |
| BMI | 45.6±3.1 | 39.9±5.8 | 0.23 |
| Waist Circumference​ | 133±22.2 | 113±14.5 | 0.65 |
| Hip Circumference | 130±17 | 131±18.4 | 0.10 |
| Insulinemia uU/mL | 12.8±5.00 | 13±6.4 | 0.93 |
| Glycemia mg/dL | 100.6±27.8 | 98±13.8 | 0.78 |
| HOMA-IR | 2.8±1.7 | 2.7±2 | 0.95 |
| HB Glycate % | 8±7.6 | 5.6±0.5 | 0.32 |
| PCR mg/dL | 8.2±11 | 5.2±4.8 | 0.44 |
| Total Cholesterol mg/dL | 168.5±36 | 170.3±34 | 0.9 |
| HDL mg/dL | 40.3±9.6 | 46.7±12.7 | 0.17 |
| LDL mg/dL | 115.4±31.4 | 110.9±30.9 | 0.72 |
| Triglycerides mg/dL | 103±42.7 | 92.8±47.3 | 0.58 |
| Menopause % subjects affected/total subjects | / | 54% | / |
| Hypertension % subjects affected/total subjects | 36.36% | 46.15% | 0.69 |
| Dyslipidemia % subjects affected/total subjects | 27.27% | 7.69% | 0.3 |
| Hepatic Steatosis % subjects affected/total subjects | 36.36% | 63.64% | 1 |
| Smoking state % subjects affected/total subjects | 63.64% | 46.15% | 0.23 |
