# Supplementary figures and images for: Acetamiprid exerts sex-specific effects on adipose tissue of subjects with severe obesity
Source: Front Toxicol. 2026 Mar 10;8:1769863. doi: 10.3389/ftox.2026.1769863 (PMC13008313; doi:10.3389/ftox.2026.1769863)

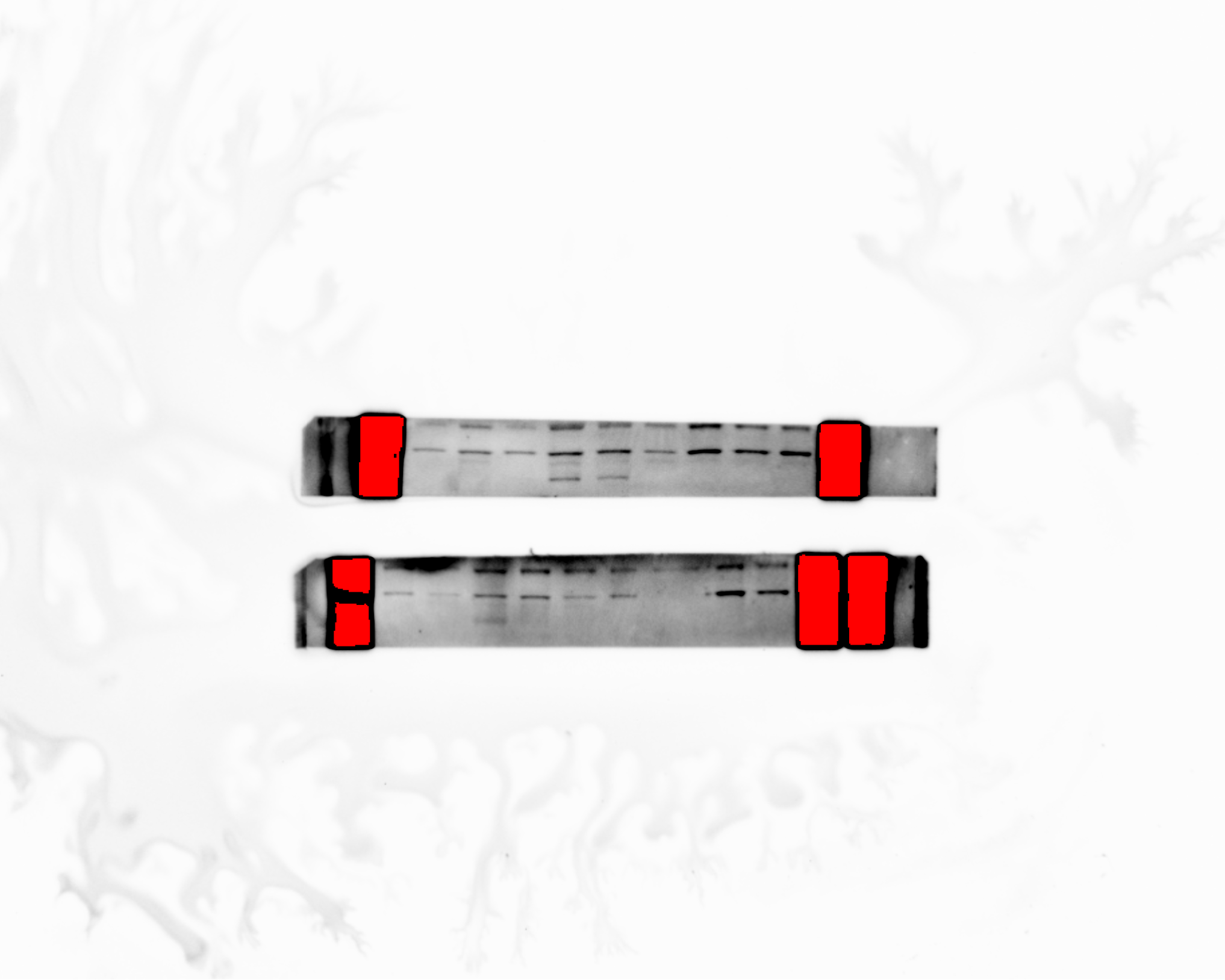

Supplement: Supplementary file 3 [file Image3.tif]

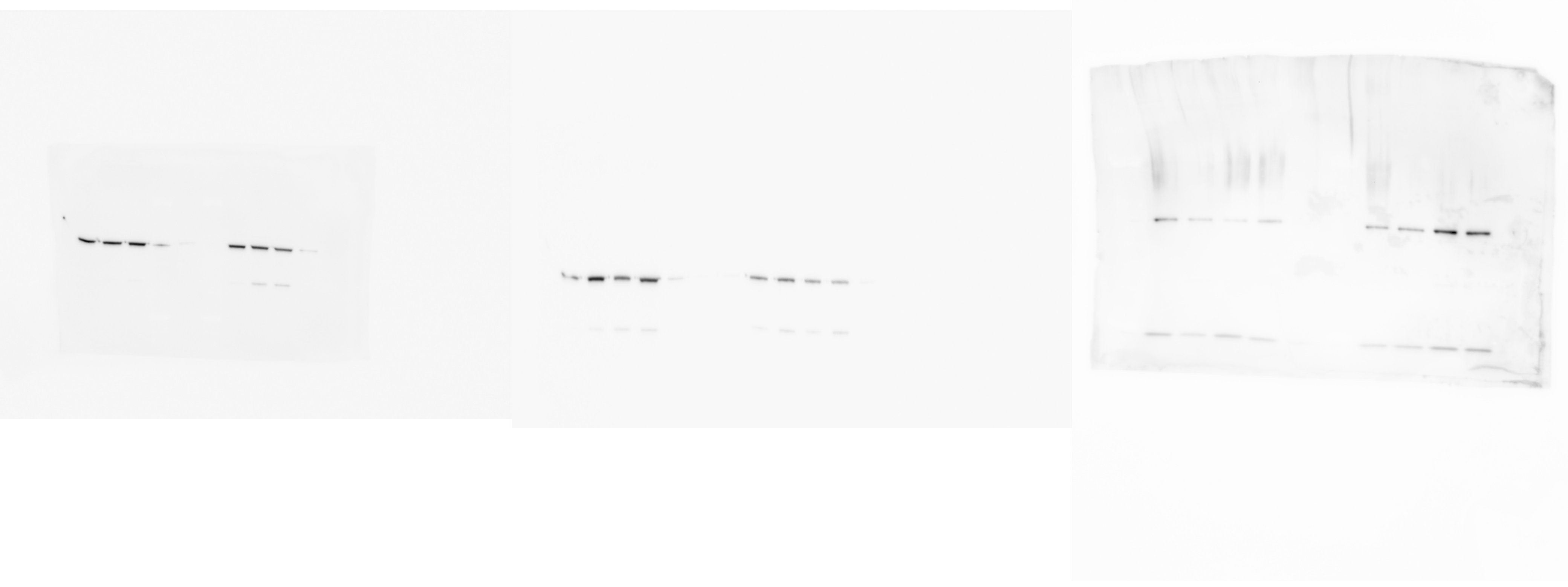

Supplement: Supplementary file 4 [file Image4.tif]

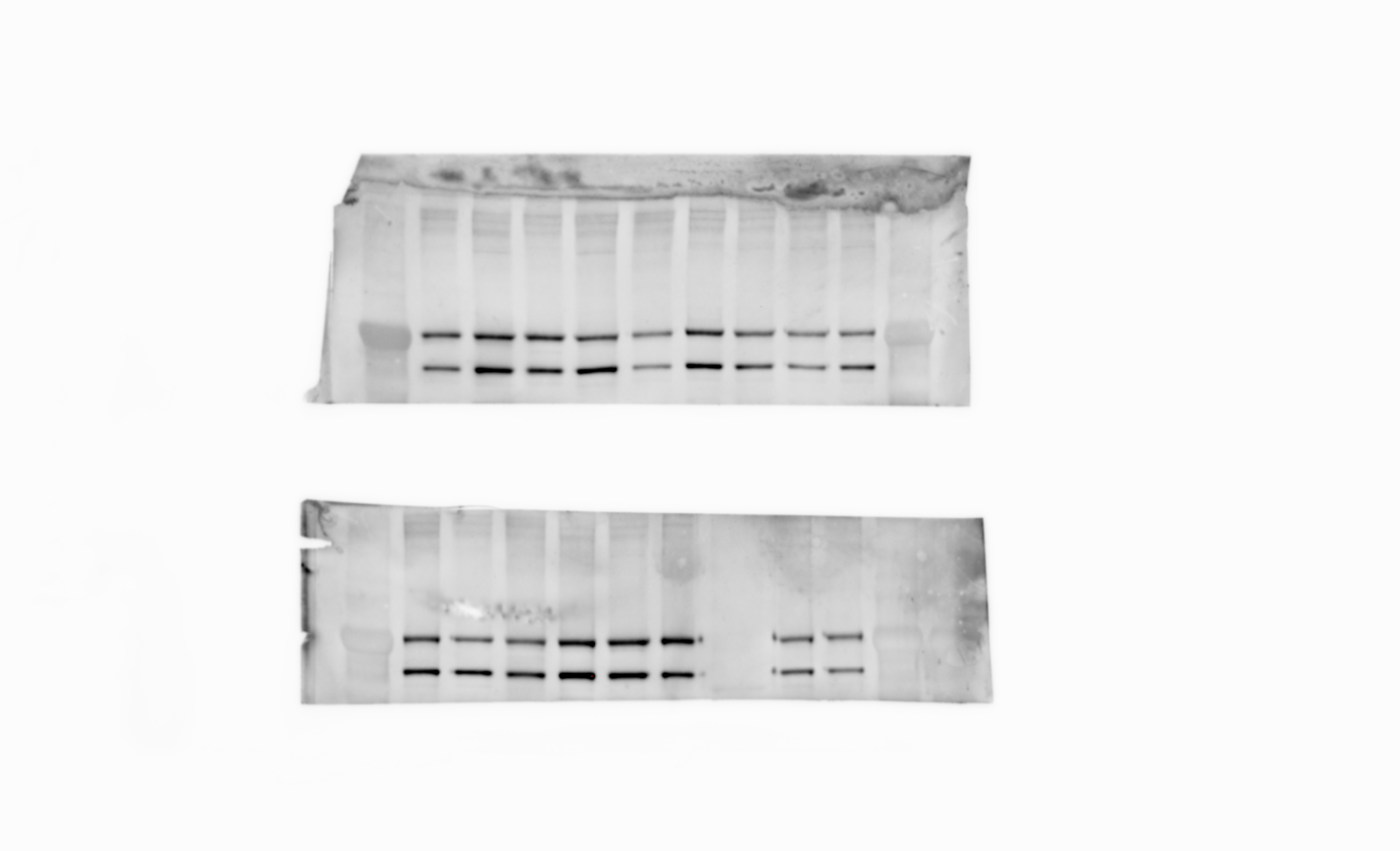

Supplement: Supplementary file 5 [file Image2.tif]

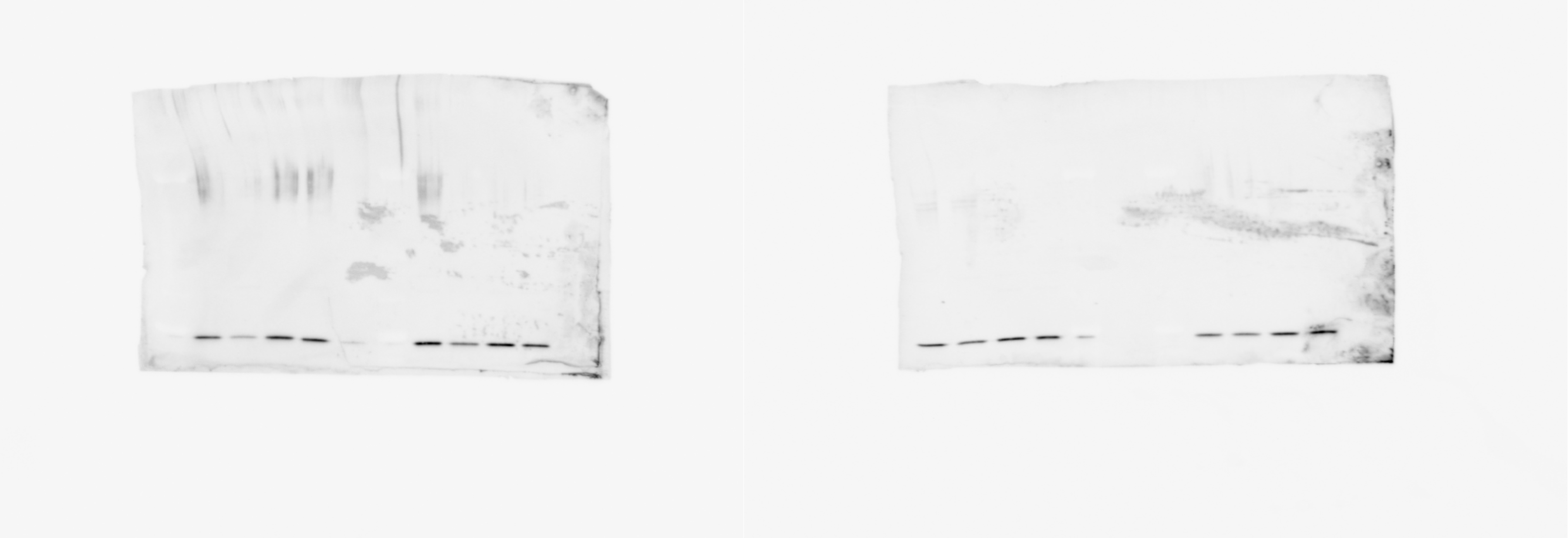

Supplement: Supplementary file 6 [file Image1.tif]
